# Supplementary material for: Mental Health Impacts of Tornadoes: A Systematic Review
Source: Int J Environ Res Public Health. 2022 Oct 22;19(21):13747. doi: 10.3390/ijerph192113747 (PMC9655757; doi:10.3390/ijerph192113747)
Supplement: Supplementary file 1 [file ijerph-19-13747-s001.zip › ijerph-1939389-supplementary.pdf]

Table S1. Assessment of risk of bias.

No. in Reference	Authors & Year	1	2	3	4	5	6	7	8	9	10	Over all bias
4	First, Jennifer M et al., 2021	Y	Y	Y	N	Y	Y	Y	Y			7/8 (Low)
15	Abel, Madelaine R et al., 2021	Y	Y	Y	N	Y	Y	Y	Y			7/8 (Low)
16	Adams, Zachary W et al., 2015	Y	Y	Y	N	Y	Y	Y	Y			7/8 (Low)
17	Bountress, Kaitlin E et al., 2017	Y	Y	Y	N	Y	Y	Y	Y			7/8 (Low)
18	Bountress, Kaitlin E et al., 2020	Y	Y	Y	Y	Y	Y	Y	Y			7/8 (Low)
19	Carbone, Eric G et al., 2017	Y	Y	Y	N	Y	Y	Y	Y			7/8 (Low)
20	Clay, Lauren A & Greer, Alex 2019	N	Y	Y	N	Y	Y	Y	Y			7/8 (Low)
21	Cohen, Joseph R et al., 2016	Y	Y	Y	N	Y	Y	Y	Y			7/8 (Low)
22	Danielson, Carla K et al., 2017	Y	Y	Y	N	Y	Y	Y	Y			7/8 (Low)
23	Evans, L. G & Oehler-Stinnett, J, 2006	N	N	Y	N	Y	N	Y	Y			4/8 (Moderate)
24	Gilmore, Amanda et al., 2018	Y	Y	Y	N	Y	N	N	Y			5/8 (Moderate)
25	Hambrick, Erin P et al., 2016	Y	N	Y	N	Y	Y	Y	Y			6/8 (Moderate)
26	Hambrick, Erin P et al., 2017	Y	N	Y	N	Y	Y	N	Y			5/8 (Moderate)
27	Hendrickson, Michelle L et al., 2018	Y	Y	Y	N	Y	Y	Y	Y			7/8 (Low)
28	Houston, J. Brian & Franken, Noah J, 2015	Y	Y	Y	N	Y	N	N	Y	N/A		5/8 (Moderate)
29	Houston, J. Brian et al., 2015	Y	Y	Y	N	Y	Y	Y	Y			7/8 (Low)
30	Knight, Laura A & Sullivan, Maureen A, 2006	N	N	N	N	Y	N	N	Y			2/8 (High)
31	Lack, Caleb W & Sullivan, Maureen A, 2008	Y	N	N	N	Y	Y	Y	Y			5/8 (Moderate)
32	Lim, Jungkyu et al., 2019	Y	N	N	N	Y	N	N	Y			3/8 (Moderate)
33	Lochman, John E et al., 2017	Y	Y	Y	N	Y	Y	Y	Y			7/8 (Low)
34	McKinzie, Ashleigh E, 2018	Y	Y	N	N	Y	N	N	N			3/8 (Moderate)
35	Paul, Lisa A et al., 2015	Y	Y	Y	N	Y	N	N	Y			5/8 (Moderate)
36	Powell, Tara M. & Bui, Tuyen, 2016	Y	Y	Y	N	Y	Y	Y	Y			7/8 (Low)
37	Prohaska, Ariane, 2020	Y	Y	Y	N	Y	Y	Y	Y			7/8 (Low)
38	Resnick, Heidi et al., 2020	Y	Y	Y	N	Y	N	N	Y			5/8 (Moderate)
39	Schroeder, Janine M and Polusny, Melissa A, 2004	N	N	N	N	Y	Y	Y	Y			4/8 (Moderate)
40	Yuan, Guangzhe et al., 2018	Y	Y	Y	N	Y	Y	Y	Y			7/8 (Low)
41	Zuromski, Kelly L et al., 2019	Y	Y	Y	N	Y	N	Y	Y			6/8 (Low)
42	Ruggiero, Kenneth J et al., 2015	Y	Y	Y	N	Y	Y	Y	Y			7/8 (Low)

Applicable

1. Was the study's target population a close representation of the national population in relation to relevant variables, e.g., age, sex, occupation? (yes = low risk, no = high risk)
2. Was the sampling frame a true or close representation of the target population? (yes = low risk, no = high risk)
3. Was some form of random selection used to select the sample, or, was a census undertaken? (yes = low risk, no = high risk)
4. Was the likelihood of non-response bias minimal? (yes = low risk, no = high risk)
5. Were data collected directly from the subjects (as opposed to a proxy)? (yes = low risk, no = high risk)
6. Was an acceptable case definition used in the study? (yes = low risk, no = high risk)
7. Was the study instrument that measured the parameter of interest (e.g., prevalence of low back pain) shown to have reliability and validity (if necessary)? (yes = low risk, no = high risk)
8. Was the same mode of data collection used for all subjects? (yes = low risk, no = high risk)

Not applicable

9. Was the length of the shortest prevalence period for the parameter of interest appropriate? (yes = low risk, no = high risk)
10. Were the numerator(s) and denominator(s) for the parameter of interest appropriate? (yes = low risk, no = high risk)

Table S2. Supporting Information: Study characteristics and overview.

No. in Ref.	Year	Title	Author(s)	Method(s)	EF scale	Tornado	Areas	Participants	Aim(s)	Result(s)
4	2021	Identifying Risk and Resilience Factors Impacting Mental Health among Black and Latinx Adults following Nocturnal Tornadoes in the U.S. Southeast	First, Jennifer M et al.	Survey	EF4	2–3 March 2020 nocturnal tornado outbreak in Middle Tennessee	i) Mental health impacts of tornadoes, ii) Risk factors and iii) Protective factors	221 Black and Latinx adult (18 years or older)	1. To Identify barriers to receiving tornado warning alerts 2. To Identify a relationship between the barriers, tornado exposure, and adverse mental health outcomes 3. To examine if resilience factors contributed to adverse mental health outcomes	1. Barriers to warning alerts had a significant and positive relationship with more tornado exposure ($\beta = 0.196$, $p < 0.05$). 2. Barriers to warning alerts was related to PTS ($\beta = 0.200$, $p < 0.01$) and depression symptoms ($\beta = 0.268$, $p < 0.001$). 3. Resilience had a significant and inverse relationship with PTS ($\beta = -0.149$, $p < 0.01$) and depression ($\beta = -0.229$, $p < 0.001$).
15	2021	Talking with children about natural disasters: Maternal acknowledgment, child emotion talk, and child posttraumatic stress symptoms.	Abel, Madelaine R et al.	Quantitative (Data analytic after Interviews)	EF5	2011 Tornado outbreak in Alabama and Joplin	i) Mental health impacts of tornadoes	Forty-nine children ages 8–12 years and their mothers	To examine the association between qualities of parent-child co-reminiscing and children's posttraumatic stress symptoms after a tornado	1. Child negative emotion words were positively associated with PTSS at lower levels of maternal support ($B=3.04$, $SE=1.07$, $p=0.007$, 95% CI [0.88, 5.20]). 2. Child positive emotion words was positively associated with PTSS at lower levels of maternal support ($B=2.14$, $SE=0.87$, $p=0.02$, 95% CI [0.38, 3.90]).
16	2015	Comorbidity of PTSD, Major Depression, and Substance Use Disorder Among Adolescent Victims of the Spring 2011	Adams, Zachary W et al.	Survey	EF5	2011 Tornado outbreak in Alabama and Joplin	i) Mental health impacts of tornadoes	2,000 adolescents aged 12 to 17 years	1. To estimate the prevalence of comorbid posttraumatic stress disorder, major depressive episode, and	1. The most common pattern of comorbidity was PTSD + MDE. 2. Approximately one-quarter of adolescents who met criteria for PTSD (26.9%) or MDE (22.7%) also met the threshold for probable SUD. 3. The most consistent predictor of each post-disaster comorbidity profile was prior exposure to traumatic events.

		Tornadoes in Alabama and Joplin, Missouri							substance use disorder<br>2. To identify risk factors for patterns of comorbidity among adolescents	4. Among 12- and 13-year-olds, girls (5.6%) endorsed a significantly higher occurrence of PTSD + MDE than boys (2.4%), X2 (1, n = 660) = 3.90, p = .048, ϕ = .08.<br>5. Male gender was associated with lower likelihood of PTSD + MDE (OR = 0.47) and MDE + SUD (OR = 0.11).<br>6. Exposure to prior traumatic events (OR = 3.29) was associated with higher odds of meeting diagnostic criteria for MDE + SUD (OR = .15) and extent of service loss (OR = 1.95) were also predictive of MDE + SUD comorbidity.
17	2017	Genetic and psychosocial predictors of alcohol use trajectories among disaster-exposed adolescents	Bountress, Kaitlin E et al.	Survey	EF5	2011 Tornado outbreak in Alabama and Joplin	i) Mental health impacts of tornadoes and<br>ii) Risk factors	2,000 adolescents aged 12 to 17 years and their caregivers	To examine how severity of disaster exposure, genetic risk, PTSD symptoms, and emotional support predict alcohol use in youth	1. Older adolescents endorsed drinking .209 more drinks a month than younger adolescents (B = .209, p < .001).<br>2. Males reported drinking .050 more drinks a month at the 12-month follow-up than females (B = .050, p < .01).<br>3. The interaction between genetic risk and severity of exposure (B = .347, p < .01) suggests that for a one unit increase in severity, individuals one standard deviation above the mean on genetic risk increased their drinking by .279 drinks per month (B = .279, p < .01).<br>4. The interaction between emotional support and PTSD symptoms (B = -.773, p < .001) suggests that for that one standard deviation below the mean on emotional support, a one unit increase in PTSD symptoms was associated with an increase of .557 drinks per month (b = .557, p < .001).
18	2020	Impact of disaster exposure severity: Cascading effects across parental distress, adolescent PTSD symptoms, as	Bountress, Kaitlin E et al.	Survey	EF5	2011 Tornado outbreak in Alabama and Joplin	i) Mental health impacts of tornadoes and<br>ii) Risk factors	1,271 adolescents (ages 12-17) and caregivers	To clarify the prospective associations among such constructs following severe disasters	1. Greater severity of disaster exposure was associated with increased parental distress and more adolescent PTSD symptoms.<br>2. Higher levels of initial parental distress were associated with more adolescent PTSD symptoms.<br>3. Family income was a significant predictor of parental distress.

19	2017	well as parent-child conflict and communication								4. More depressive symptoms were predictive of more parent distress, more adolescent PTSD symptoms, and poorer parent-child conflict communication.<br>5. Adolescent alcohol use was largely not associated with constructs of primary interest at any time point, with the exception of baseline adolescent PTSD symptoms.
19	2017	Effects of optimism on recovery and mental health after a tornado outbreak	Carbone, Eric G et al.	Survey	EF5	The April 2011 tornado outbreak in Mississippi and Alabama	iii) Protective factors	3,216 participants who were 18 years or older	To assess the effect of dispositional optimism on post-disaster recovery and mental health	1. A significant positive association between optimism and recovery, but optimism was more strongly related to the quality-of-life change for significant levels of home damage ($p < .0001$, $\beta = .40$).<br>2. Optimism was negatively associated with the level of symptomatology of depression ($p < .0001$), anxiety ($p < .0001$) and PTSD ($p < .0001$).<br>3. Extent of home damage was positively associated with depressive symptoms ($p < .0001$), anxiety symptoms ($p < .0001$) and PTSD symptoms ($p < .0001$).
20	2019	Association between long-term stressors and mental health distress following the 2013 Moore tornado: a pilot study	Clay, Lauren A & Greer, Alex	Survey	EF5	Moore, Oklahoma in 2013	ii) Risk factors	71 surveys were returned of 750 households	To examine the influence of stressors including resource loss and debt on mental health in a sample of households	1. $\chi 2$ and Fisher's exact analysis of disaster exposure measures with the outcome mental health showed that debt ($p<0.01$) and resource loss ($p<0.001$) were independently associated with mental health.<br>2. In unadjusted logistic regression, high resource loss was a risk factor for poor mental health. For each one unit increase in resource loss summary score, respondents are three percent more likely to report poor mental health (OR 1.03, 95% CI 1.01, 1.06) and respondents that reported taking on debt due to the tornadoes were greater than five times more likely to report poor mental health (OR 5.67, 95% CI 1.60, 20.13).

21	2016	Distress Tolerance and Social Support in Adolescence: Predicting Risk for Internalizing and Externalizing Symptoms Following a Natural Disaster	Cohen, Joseph R et al.	Survey	EF5	2011 Tornado outbreak in Alabama and Joplin	i) Mental health impacts of tornadoes	352 adolescents aged 12–17 years	To examine the interactive relation between behavioral distress tolerance and perceived social support	1. For depression and PTSD, age, sex, race, and completing the intervention did not influence the relation between DT/PSS and symptoms ($p > .05$).<br>2. As with depression, the interaction between DT and PSS predicted prospective PTSD symptoms was significant ($B = −0.0002$; $SE = 0.0001$; $t(195) = −2.08$, $p = .04$; reffect size = 0.15).<br>3. PSS ($B = 0.0039$; $SE = 0.0018$; $t(139) = −2.20$, $p = .03$; $r_{effect\ size}=0.18$), but not DT ($B = −0.0001$; $SE = 0.0001$; $t(130) = −0.79$, $p = .43$; $r_{effect\ size}=0.07$), predicted substance use, such that lower levels of PSS predicted elevated levels of substance use.
22	2017	Clinical Decision-Making Following Disasters: Efficient Identification of PTSD Risk in Adolescents	Danielson , Carla K et al.	Survey	EF5	2011 Tornado outbreak in Alabama and Joplin	i) Mental health impacts of tornadoes and<br>ii) Risk factors	2,000 adolescent-parent dyads	To assess individual, interpersonal, and event-related vulnerability factors to determine which indices were most accurate in forecasting PTSD	1. Females and older adolescents were more likely to report a diagnosis of PTSD since the natural disaster ($p < .05$).<br>2. Distress tolerance and risk-taking propensity did not discriminate PTSD significantly better than chance ($p > .05$). Furthermore, disaster impact (AUC=.62) and lifetime substance abuse (AUC=.68) were deemed poor predictors (i.e., AUC <.70) of PTSD.<br>3. Lifetime depression predicted PTSD diagnosis significantly better than trauma history ($z=7.07$, $p<.001$), social support ($z=10.00$, $p<.001$), and family conflict ($z=6.04$, $p<.001$).<br>4. Trauma history outperformed social support ($z=2.34$, $p<.05$).<br>5. The interaction between social support and family conflict ($B=.03$, $SE=.02$; $W=4.59$, $p=.03$) was the only significant interaction between the indicator variables (all other $ps>.10$).
23	2006	Structure and prevalence of PTSD symptomology in children who	Evans, L. G & Oehler-Stinnett, J	Survey	EF5	Tornadoes in Oklahoma	i) Mental health impacts of tornadoes and<br>ii) Risk factors	152 children, Ages ranged from 6 to 12 years	To evaluate the relationship between children's fear during the tornado and	1. How scared the child was during the tornado was significantly correlated with Avoidance, Re-experiencing, Interpersonal Alienation, and the total Score.

		have experienced a severe tornado							damage to their school and PTSD	2. Whether the school was damaged was related to Avoidance, Foreshortened Future, and the total Score.<br>3. Whether the child's home was damaged and how close the child was to the tornado were not significantly related to the OSU PTSD-CF.
24	2018	A Longitudinal Examination of Interpersonal Violence Exposure, Concern for Loved Ones During a Disaster, and Web-Based Intervention Effects on Posttraumatic Stress Disorder Among Adolescent Victims of the Spring 2011 Tornadoes	Gilmore, Amanda et al.	A secondary data analysis	EF5	2011 Tornado outbreak in Alabama and Joplin	ii) Risk factors	2,000 adolescents	To examine adolescents' history of IPV and concern for loved ones during the disaster as moderators of treatment outcomes 4- and 12-months after receiving the BBN intervention	1. The main effects model revealed a significant association between IPV exposure and PTSD symptoms and an association between gender and PTSD at baseline.<br>2. A significant interaction was found between time, treatment condition (BBN vs. control), and concern for loved ones on PTSD symptoms. Probing of this interaction revealed that among those who were not concerned for loved ones during the disaster, there was no main effect of change over time for adolescents in the BBN condition or the control condition, control: $b = -0.03$, $p = .338$, odds ratio (OR) = 0.97; BBN: $b = -0.02$, $p = .120$, OR = 1.02.<br>3. Among those who were concerned for loved ones, a different pattern of results emerged. Those in the BBN condition experienced a significant decline in PTSD symptoms over time ($b = -0.02$, $p = .041$, OR = 0.98), whereas those in the control condition did not ($b = 0.02$, $p = .255$, OR = 1.02).
25	2016	Interview and recollection-based research with child disaster survivors: Participation-related changes in emotion and perceptions of participation	Hambrick, Erin P et al.	An in-person interview and phone surveys	EF5	2011 Tornado outbreak in Alabama and Joplin	i) Mental health impacts of tornadoes and<br>ii) Risk factors	Fifty 8- to 12-year-old children	To evaluate concurrent and persistent risks and benefits associated with preadolescent disaster survivors' participation in recollection-based disaster research	Posttraumatic stress symptom severity, tornado exposure, and age were largely unrelated to child-reported emotions and perceptions of research. Results indicate that carefully planned and executed disaster-related research that includes children providing recollections can be conducted with preadolescents with little risk and some benefit.

26	2017	Recalling a Devastating Tornado: Qualities of Child Recollections and Associations with Mental Health Symptoms	Hambrick, Erin P et al.	survey	EF5	2011 Tornado outbreak in Alabama and Joplin	i) Mental health impacts of tornadoes and ii) Risk factors	Fifty 8- to 12-year-olds and their mothers	To explore links between indicators of children's cognitive and emotional processing of trauma in recollections about a tornado and concurrent mental health symptoms	1. Correlations between mental health symptoms, tornado exposure, and child processing variables were found, two hierarchical linear regressions were conducted.<br>2. When controlling for age, gender, verbal ability, whether or not the child received tornado-related mental health treatment, frequency of event rehearsal, family income, and tornado exposure, child processing variables were significantly associated with mental health symptoms. Specifically, Coherence (B=0.275, t = 3.607, p = .001), Positive Emotion (B = 12.425, t = − 3.137, p = 003), and Resolutions (B = − .598, t=−2.507, p=.017) were associated with mental health symptoms.<br>3. Whether or not a child had received tornado-related mental health treatment was the only other significant variable in the regression (B=−6.94, t=−2.44, p=.019).
27	2018	Caregiver–adolescent co-reminiscing and adolescents' individual recollections of a devastating tornado: Associations with enduring posttraumatic stress symptoms	Hendrickson, Michelle L et al.	Individual interviews	EF4	Tuscaloosa, Alabama in 2011	ii) Risk factors	122 female caregiver and adolescent (aged 12~17) dyads	To evaluate the relation among caregiver–adolescent communication patterns and youth PTSS years after a tornado	1. There was a significant effect of caregiver egocentrism on youth PTSS, t (118) = 2.02, p = .045, a significant effect of caregiver egocentrism on youth coherence, t (118) = 3.26, p = .001, and a significant effect of youth coherence on youth PTSS, t (118) = 2.51, p = .01. Further, the indirect effect of egocentrism on youth PTSS through coherence had a 95% CI that did not contain zero [0.07, 2.18].<br>2. There was a nonsignificant effect of youth negative personal impact on youth PTSS, t (115) = 0.97, p = .33. However, the interaction of negative personal impact and coherence was significant, t (115) = 2.39, p = .018.
28	2015	Disaster interpersonal communication and posttraumatic stress following	Houston, J. Brian & Franken, Noah J	Survey	EF5	2011 Tornado outbreak in Alabama and Joplin	ii) Risk factors	380 Adult residents at least 18 years old	To understand how sociodemographics, disaster experience, and disaster reactions	1. Female talked with family and friends (b = .12, p < .019) and neighbors (b = .11, p < .039) about the tornado more frequently than male participants.<br>2. Participants with direct exposure to the tornado talked about the event with neighbors more frequently (b = .15, p = .004) than participants

		the 2011 Joplin, Missouri, Tornado						influence the amount of disaster talk among residents after a tornado	without direct exposure, and participants who knew someone who died in the tornado attended more community meetings about the tornado (b = .22, p < .001) than participants who did not know someone who died in the storm.<br>3. Participants with more PTS symptoms discussed the tornado with friends and family (b = .11, p = .045) and neighbors (b = .12, p = .039) more frequently and attended more community meetings about the tornado (b = .18, p = .001) than participants with fewer PTS symptoms.
29	2015	2011 Joplin, Missouri Tornado Experience, Mental Health Reactions, and Service Utilization: Cross-Sectional Assessments at Approximately 6 Months and 2.5 Years Post-Event	Houston, J Brian et al.	Survey	EF5	2011 Tornado outbreak in Alabama and Joplin	i) Mental health impacts of tornadoes,<br>ii) Risk factors and<br>iii) Protective factors	438 Joplin adults	To examine an association between residents' post-tornado experiences, PTS, depression, mental health service, sociodemographic and mental health effects after a tornado	1. Participants experiencing high tornado impact were more likely than participants experiencing low tornado impact to report probable PTSD and more likely to report current depression.<br>2. Participants with high levels of social support were less likely than participants with low levels of social support to report probable PTSD (OR, 0.36; 95% CI, 0.18-0.73) and current depression (OR, 0.35; 95% CI, 0.17-0.75).<br>3. The majority of individuals with probable PTSD reported speaking with a counselor or mental health professional (83.42%) and a religious professional (85.91%) not at all or very little.
30	2006	Preliminary Development of a Measure to Assess Children's Trauma Attributions	Knight, Laura A & Sullivan, Maureen A	Survey	N/A	A series of tornadoes in Oaklahoma (authors did not specify the tornado data)	i) Mental health impacts of tornadoes	52 children in age from 8 to 12 years	To assess natural disaster-related attributions in children	1. Significant gender differences were found on two TAC subscales, with girls scoring higher on the Search for Meaning and Expectations of Recurrence subscales (t[49] = -2.561, p = .014; t[49] = -2.111, p = .040, respectively).<br>2. A significant relationship also emerged between total RI score and degree of child-reported tornado exposure as measured by the TEQ, r (52) = .57, p = .05.
31	2008	Attributions, coping, and exposure as predictors of	Lack, Caleb W & Sullivan,	Survey	EF3	Oklahoma, 2001	i) Mental health impacts of tornadoes and	Children in Grades 3 through 6 and their	To examine the predictive ability of three factors (attributions,	1. A series of correlational analyses were conducted to determine the relationship between posttraumatic distress and the level of exposure the child had to the tornado. Statistically significant

		long-term posttraumatic distress in tornado-exposed children.	Maureen A				ii) Risk factors	parents, a total of 102 children and 96 parents	coping strategies, and degree of exposure to the traumatic event) related to posttraumatic distress	relationships were found between the total RI score and parent report of both how scared the child was during the tornado and how worried he or she had been since the tornado, r(98) = .238, p = .018, and r(98) = .240, p = .017, respectively. A significant relationship was also found between total RI score and the child's report of how scared he or she was during the tornado, r(102) = .480, p < .001.<br>2. A statistically significant correlation was found between RI severity score and total score on the TAC, r(102) = .714, p < .001.
32	2019	Individuals' religiosity and emotional coping in response to disasters	Lim, Jungkyu et al.	Focus group interview and survey	N/A	Non-specific	i) Mental health impacts of tornadoes and<br>iii) Protective factors	- Focus group: 77 Residents of the Southeastern United States<br><br>- Survey: 1,484 Southeast U.S. residents	To examine how individuals' religiosity and emotions affect coping in response to disasters	1. Only fear and hope were significant among various emotions tested with religiosity, the researchers reduced the model. Results from the reduced model indicate that fear, Exp(B) = 1.393, p < 0.001, and hope, Exp(B) = 1.307, p < 0.01, were significant predicting variables for taking physical action, controlling for demographics.<br>2. Since religiosity was one of few significant variables, the researchers reduced the model. Results from the reduced regression model indicate that religiosity, Exp(B) = 1.419, p < 0.001, significantly predicted physical action taking, controlling for demographics.
33	2017	Pre-Post Tornado Effects on Aggressive Children's Psychological and Behavioral Adjustment Through One-Year Postdisaster	Lochman, John E et al.	Survey	EF4	Tuscaloosa County, Alabama. 2011	i) Mental health impacts of tornadoes and<br>ii) Risk factors	360 children in Grades 4–6	To examine how levels of exposure to a tornado and children's characteristics influenced the behavioral and psychological adjustment of children 1-year post-disaster	1. Children's pre-tornado anxiety level was found to moderate their experience of the degree of exposure to the disaster. When children had high baseline anxiety and they had not been afraid of dying during the tornado, they were likely to have greater declines in parent-rated aggressive and internalizing behavior outcomes across.<br>2. The degree of exposure to the tornado that children experience negatively influences at-risk children's emotional adjustment through the 1-year follow-up.

34	2018	In their own words: disaster and emotion, suffering, and mental health	McKinzie, Ashleigh Elain	Participant observation, interviews, and archival work	EF5	2011 Tornado outbreak in Alabama and Joplin	i) Mental health impacts of tornadoes	162 People who were affected by the tornado and local leaders and volunteers	To explore emotions, trauma, and mental health issues residents experienced after tornadoes	Four common themes:<br>1) Emotions in immediate aftermath<br>2) Relationship strain<br>3) Mental health problems<br>4) Emotions in long-term recovery<br>were found.
35	2015	Mental Health Among Adolescents Exposed to a Tornado: The Influence of Social Support and Its Interactions With Sociodemographic Characteristics and Disaster Exposure	Paul, Lisa A et al.	Survey	EF5	2011 Tornado outbreak in Alabama and Joplin	ii) Risk factors	2,000 adolescents aged 12 to 17 years and their caregivers	To assess adolescents' social support from family and peers and its potential interaction with the disaster-related environment and sociodemographic characteristics on post-tornado distress	1. Youth with low income experienced a greater number of PTSD symptoms, M = 3.16, 95% CI [2.79, 3.54], and depressive symptoms, M = 1.77, 95% CI [1.56, 1.98], than youth in households reporting high income.<br>2. Adolescent girls reported greater experienced tornado severity (M = 3.18, SD = 0.10) than boys (M = 3.10, SD = 0.10), t(1966) = 2.59, p = .010. and reported experiencing a greater number of PTSD symptoms (M = 2.83, SD = 3.62) than boys (M = 2.12, SD = 3.05), t(1997) = 4.69, p < .001.<br>3. Adolescent girls also reported a greater number of post tornado depressive symptoms (M = 1.56, SD = 2.13) than boys (M = 1.13, SD = 1.70), t(1996) = 4.50, p < .001.<br>4. Lower levels of social support (β = −.57, p < .001), greater experienced tornado severity (β =.15, p < .001), lower household income (β = .13, p = .013), female sex (β = −.67, p < .001), and older age (β = .13, p = .002) were associated with greater PTSD symptoms.<br>5. less social support (β = −.35, p < .001), lower household income (β = −.10, p = .002), greater experienced tornado severity (β = .07, p < .001), female sex (β = .38, p < .001), and older age (β = .10, p < .001) were associated with greater depressive symptoms.
36	2016	Supporting social and emotional skills after a	Powell, Tara M &	Mixed method	EF5	Moore, Oklahoma	iv) Mental health interventions	110 students ranged between 11	To explore the impact of a school-based	1. The first paired-samples t-test indicated that there was a significant difference in the scores for the Journey of Hope group on communication

		disaster: Findings from a mixed methods study.	Bui, Tuyen	(survey and interview)				and 15 years old.	psychosocial curriculum entitled Journey of Hope	management t(53) = -1.96, p = .05, while there was no significant difference for the control group t(48) = .89, p = . 37.<br>2. A paired-samples t test was then run on prosocial behaviors, indicating a significant difference for the JoH group t(59) = -4.59, p = .000, whereas there were no significant differences for the control group t(48) = .39, p = .69.<br>3. Significant differences were not detected on problem-solving F(1,97) = .42, p = .517; d = .11, personal development F(1,108) = .422, p = .517; d = .04, or general self-efficacy F(1,107) = .174, p = .678; d = .08.
37	2020	Still struggling: intersectionality, vulnerability, and long-term recovery after the Tuscaloosa, Alabama USA Tornado	Prohaska, Ariane	In-depth interviews	EF4	Tuscaloosa, Alabama, 2011	i) Mental health impacts of tornadoes and<br>ii) Risk factors	29 tornado survivors	To understand how pre-tornado vulnerabilities and social locations affected tornado survivors' recovery 4 to 5 years after a tornado	1. Latina women experienced very specific stressors related to the intersections of language barriers, citizenship, ethnicity, and social class.<br>2. Being a renter, a person of color, and lacking insurance pre-storm were major barriers to financial recovery in the long term for the participants in this study.<br>3. The pre-storm stressors of poverty, anxiety, and depression, then, exacerbated the emotional distress of the tornado.
38	2020	Prior Interpersonal Violence Exposure and Experiences During and After a Disaster as Predictors of Posttraumatic Stress Disorder and Depression Among Adolescent Victims of the	Resnick, Heidi et al.	Survey	EF5	2011 Tornado outbreak in Alabama and Joplin	ii) Risk factors	2,000 adolescents aged 12 to 17 years and their caregivers	To examine the relative roles of demographics, disaster exposure, and specific prior classes of traumatic events (disaster, accident, IPV) as predictors of symptoms of PTSD and depression	1. Gender was negatively associated with both PTSD and MDD, such that males had roughly half the number of PTSD and MDD symptoms than did females. Age also emerged as significantly positively related to MDD symptoms only, though this effect was small (IRR 1.07, 95% confidence interval [CI] = [1.01, 1.12]).<br>2. Individuals who experienced a physical injury during the tornadoes had nearly double the number of PTSD and MDD symptoms endorsed compared with those who did not experience physical injury.

		Spring 2011 Tornadoes								3. Concern about others during the tornadoes also emerged as significantly positively related to PTSD symptoms only (IRR = 1.40, 95% CI = [1.14, 1.72]).<br>4. Prevalence of past month PTSD was significantly higher among those reporting history of IPV (5.50% vs. 0.60%), $\chi 2$ (1, N = 1,926) = 47.14, p < .001, as was prevalence of past month depression (6.30% vs. 0.60%), $\chi 2$ (1, N = 1,978) = 56.32, p < .001.
39	2004	Risk Factors for Adolescent Alcohol Use Following a Natural Disaster	Schroeder, Janine M & Polusny, Melissa A	Survey	F-3 and F-4	On 29 March 1998, tornadoes in four rural southern Minnesota counties	i) Mental health impacts of tornadoes and<br>ii) Risk factors	256 adolescents in grades 10-12	To identify potential risk factors for alcohol use in an adolescent exposed to a tornado	1. Age, prior alcohol-related negative consequences, and prior trauma history significantly predicted adolescents' peak alcohol use six months following the disaster.<br>2. Only the extent of prior trauma history and total post-traumatic symptomatology in response to the disaster were significant predictors of adolescents' reporting an increase in the amount of alcohol consumed since the disaster. However, odds ratios of 1.26 and 1.03, respectively, shows little change in the likelihood of reporting increased drinking following the disaster on the basis of a one unit change in prior trauma history and PTSD symptomatology.<br>3. Demographic variables, prior alcohol-related negative consequences, and perceived life threat during the disaster were not significantly associated with an increase in the quantity of alcohol consumed following the tornado among this sample of adolescents.
40	2018	Resilience, Posttraumatic Stress Symptoms, and Posttraumatic Growth in Chinese Adolescents After a Tornado: The	Yuan, Guangzhe et al.	Survey	EF4	2016 Tornado in Yancheng, Jiangsu in	i) Mental health impacts of tornadoes and<br>iii) Protective factors	247 Chinese adolescents from two middle schools	To investigate a relationship between resilience, PTSD symptoms, and post-traumatic growth in adolescents by considering the	1. Resilience is associated with increased level of perceived social support, decreased avoidance, decreased hyper, and an increased level of PTG.<br>2. Further, as the level of perceived social support increased, the level of PTG also increased, and the level of intrusion decreased. Perceived social support significantly mediated the relationship between resilience and PTSD symptoms (with the indirect effect = −0.061, 95% CI = −0.103 to −0.021).

		Role of Mediation Through Perceived Social Support							role of perceived social support after a tornado	3. In addition, perceived social support also significantly mediated the relationship between resilience and PTG (with the indirect effect = 0.094, 95% CI = 0.064–0.140).
41	2019	Suicidal ideation among adolescents following natural disaster: The role of prior interpersonal violence	Zuromski, Kelly L et al.	Survey	EF5	2011 Tornado outbreak in Alabama and Joplin	i) Mental health impacts of tornadoes	2,000 adolescents	To examine an association between prior traumatic events, disaster exposure, and current mental health symptom, and suicidal ideation of residents after a tornado	1. PTSD symptoms were associated with presence of suicidal ideation (b = .11, p < .05; OR = 1.12, 95% CI [1.03, 1.22]). Similar results were found for symptoms of MDD (b = .52, p < .001; OR = 1.68, 95% CI [1.42, 1.98]).<br>2. History of IPV exposure was more robustly related to suicidal ideation compared to PTSD and MDD symptoms, such that the odds of endorsing suicidal ideation were nearly two and half times larger for those with IPV history than for those without (b = .885, p < .01; OR = 2.42, 95% CI [1.37, 4.30]).<br>3. Prevalence of suicidal ideation since the tornadoes was significantly higher among those reporting history of IPV versus those who did not report IPV history (11.30% vs. 1.80%), $\chi 2$ (1, N = 1977) = 81.93, p < .001.
42	2015	Web Intervention for Adolescents Affected by Disaster: Population-Based Randomized Controlled Trial	Ruggiero, Kenneth J et al.	Survey	EF5	2011 Tornado outbreak in Alabama and Joplin	iv) Mental health interventions	2,000 adolescents	To assess the efficacy of Bounce Back Now, a Web-based intervention for disaster-affected adolescents and their parents	Intent-to-treat analyses revealed time * condition interactions for PTSD symptoms (B = -0.24, SE = 0.08, p < .01) and depressive symptoms (B = -0.23, SE = 0.09, p < .01). Post hoc comparisons revealed fewer PTSD and depressive symptoms for adolescents in the experimental versus control conditions at 12-month follow-up (PTSD: B = -0.36, SE = 0.19, p = .06; depressive symptoms: B = -0.42, SE = 0.19, p = 0.03). A time * condition interaction also was found that favored the BBN versus BBN þ parent self-help condition for PTSD symptoms (B = 0.30, SE = 0.12, p = .02) but not depressive symptoms (B = 0.12, SE = 0.12, p = .33).
